# Parental Adverse Childhood Experiences (ACEs) in an Early Childhood Mental Health Outpatient Clinic in Germany: Prevalence and Associations with Child Psychiatric Diagnoses

**DOI:** 10.3390/children12101420

**Published:** 2025-10-21

**Authors:** Franziska Laqua, Eva Möhler, Jens Joas, Frank W. Paulus

**Affiliations:** 1 Department of Child and Adolescent Psychiatry, Saarland University, 66123 Saarbrücken, Germany; s8frlaqu@stud.uni-saarland.de (F.L.); frank.paulus@uks.eu (F.W.P.); 2 Department of Child and Adolescent Psychiatry, Saarland University Hospital, 66421 Homburg, Germany

**Keywords:** ACE, adverse childhood experiences, child, transgenerational transmission, DC:0–5

## Abstract

**Highlights:**

**What are the main findings?**

- Parents of preschool children in psychiatric care reported high rates of ACEs, with 30.2% (*n* = 35) experiencing ≧ 4 ACEs—considerably higher than in the general population.
- The most common ACEs were parental separation, household mental illness, and emotional abuse.
- Parental neglect ACEs were significantly associated with adjustment disorder in children.

**What is the implication of the main finding?**

- Intergenerational effects of ACEs may contribute to early psychiatric symptoms in preschoolers, particularly adjustment disorders. Screening for parental ACEs in child psychiatry could help identify at-risk families and develop early intervention strategies.

**Abstract:**

Parental adverse childhood experiences (ACEs) are linked to negative outcomes in children, including emotional and behavioral problems, developmental delays, and higher risk for psychopathology. Most research focuses on school-aged children or community samples, with few studies examining preschool-aged children in child psychiatric care. Understanding parental ACEs in this population is crucial, as early childhood is a sensitive developmental period, and intergenerational effects may be particularly pronounced in children already presenting with psychiatric symptoms. **Background/Objectives**: The goal of this study was to analyze how parents of patients in an early childhood (0–5.9 yrs) mental health outpatient clinic differ from the general population in terms of the frequency of ACEs. In addition, we investigated the connection between mental health disorders in young children and the specific ACE scores of their parents. **Methods**: A total of 116 caregivers (34.45 years (SD = 5.28)) and their children (71.6% boys, 28.4% girls) at an average age of 3.99 years (SD = 1.35, range = 0.31–5.95) were included in the analysis. The legal guardians completed the 10-item ACE questionnaire. The young children were diagnosed as part of outpatient treatment using the DC:0–5 classification system. We analyzed the ACE scores and diagnoses descriptively and in comparison to a community sample. **Results**: An average value of 2.38 parental ACEs was reported by our sample, and 68.1% (*n* = 79) reported at least one ACE. The high-risk group with four or more ACEs comprised 30.2% (*n* = 35). The most common diagnosis in young children was the Disorder of Dysregulated Anger and Aggression of Early Childhood, followed by global developmental delay. Adjustment disorder was third in terms of frequency. Among the examined child psychiatric diagnoses, adjustment disorder showed a significant correlation with parents being affected by the ACE category of neglect (OR = 2.54; 95% CI: 1.012–6.369; *p* = 0.047). **Conclusions**: Parents who presented their children at an early childhood mental health outpatient clinic reported significantly more ACEs as compared to representative data on ACEs in adulthood. These results highlight the need for further studies with larger samples to enable a more in-depth analysis of the general intergenerational transmission processes and the differential transmission of specific ACEs to specific diagnoses in preschool-aged children.

## 1. Introduction

Adverse childhood experiences (ACEs) have been found to be significant risk factors that increase the likelihood of adverse health outcomes, such as alcoholism, drug abuse, depression, and suicide attempts. This was first described by Felliti et al. 1998, whose study examining adverse childhood experiences and their link to difficulties in adulthood formed the foundation for further research in this field [1].

The term adverse childhood experiences (ACEs) describes various forms of stressful experiences in childhood—such as abuse, neglect, or family dysfunction—that have been shown to be associated with long-term negative effects on health and well-being in adulthood [2]. A total of four or more ACEs is associated with increased behavioral problems in children [3]. In addition, higher exposure to adverse childhood experiences is associated with greater demand for coping mechanisms for mood regulation. Affected people are more likely to live unhealthily and develop a life with various risk factors such as smoking, alcoholism, drug abuse, depression, and suicide attempts, which, in turn, lead to related diseases such as diabetes, chronic bronchitis, and hepatitis. This behavior is unfavorable for the cardiovascular system. This vicious circle contributes to the development of multiple risk factors and increased health risks that should be recognized as the basic causes of morbidity and mortality in adulthood [1]. Research focuses not only on the effects of ACEs on later adult life but also on the transmission of stressful childhood experiences from parents to their own children. Multiple studies have focused especially on mothers and maternal childhood maltreatment, which was directly associated with higher levels of maladaptive infant socioemotional symptoms [4]. Furthermore, children showed more psychosocial challenges and were more likely to have difficulties in life such as conduct problems, hyperactivity, emotional and peer problems when their mother reported high ACE scores [5]. A systematic review included 16 studies and showed that maternal ACEs were significantly associated with externalizing disorders in children. In addition, these children had more difficulties with anxiety and depression [6].

A study published in 2018 analyzed the risks of a transgenerational effect of ACEs. To this end, stressful childhood experiences among parents and behavioral problems among their children aged 0–17 were examined [3]. A parental ACE score of four or higher significantly increased the risk of the affected children exhibiting behavioral problems. This was further supported by a scoping review Zhang et al. (2023) that demonstrated associations between parental ACEs and multiple aspects of children’s outcomes, including physical health, mental health, cognitive development, and social functioning [7]. A meta-analysis by Racine et al. (2023) showed that higher parental ACE scores were associated with greater mental health difficulties in children in general and with internalizing (anxiety, sadness, social withdrawal) and externalizing (hyperactivity, aggression) difficulties in particular [8]. Consistent with this, studies have shown that children who face more challenges are more likely to suffer from externalizing and internalizing behaviors [9]. Studies such as those by Folger et al. (2018) and Schickedanz et al. (2018) have shown increased rates of emotional and behavioral problems (e.g., sadness, anxiety, withdrawal) in children of parents with ACEs—symptoms that could be subsumed under adjustment disorders in childhood [3,10]. Witt et al. examined ACEs in the German population in a 2019 study. Here, 43.7% of participants reported having experienced at least one stressful childhood experience, while 8.9% classified themselves in the category of four ACEs or higher. According to the study, participants in this category had increased risks of depression, physical aggression, and impaired life satisfaction [2]. Early childhood represents a particularly sensitive period during which effects of parental adversity are thought to operate through a combination of biological and caregiving pathways [11]. Maternal caregiving has been shown to affect children’s gene expression, such as stress regulation and early attachment. For instance, oxytocin receptor signaling provides a possible mechanism for intergenerational transmission of risk in adverse environments [12]. Childhood maltreatment exacerbates these effects by producing long-term alterations in endocrine and immune function. These effects are reflected in pregnant women and the maternal-placental-fetal stress biology which shapes fetal neurodevelopment [13]. This leads to a change in epigenetic mechanisms such as DNA methylation and miRNA regulation in the offspring. These alterations can impact cognitive and emotional development and increase vulnerability to neurodevelopmental and psychiatric disorders. Comprehensive reviews further support that parental exposure to maltreatment increases the likelihood of adverse outcomes in offspring through psychosocial, behavioral, and biological pathways, with early caregiving, attachment quality, and maternal stress physiology converging to modulate intergenerational risk [14].

To date, although several reviews have synthesized the literature on parental ACEs, studies specifically assessing parental ACEs in the context of preschool children receiving mental health care are still scarce. One study in a patient group aged six years and above described significantly higher parental ACE scores compared to community samples [15]. Therefore, in the present study, we investigated whether the legal guardians of patients attending an early childhood mental health outpatient clinic (aged below six years) also differed from the general population in terms of the frequency of ACEs.

A second question examined whether parents transgenerationally pass on the specific type of ACEs from their childhood to their own children in the form of specific diagnoses. Since the effects of ACEs on the children of those affected (and especially on preschool-aged children) have not yet been sufficiently studied, our study serves to record the frequency of ACEs among parents of preschool-aged children (0 to 5 years) with mental health issues. To the best of our knowledge, this is one of the first studies to examine the transgenerational transmission of parental ACEs to preschool children in an early childhood mental health clinic. Early childhood deserves particular attention, as it represents a period of heightened neurobiological and psychological vulnerability. During this time, foundational brain circuits underlying cognition, emotion, attachment, and stress regulation are developing, making them especially susceptible to environmental influences, including parental ACEs [16,17].

## 2. Materials and Methods

Participants. The data used in this study were collected locally in Homburg/Saar by the early childhood mental health outpatient clinic (a special outpatient clinic for infants, toddlers, and preschool children aged 0–5 years). This local study formed part of the multicentric study ‘Developmental psychiatry: diagnostic challenges Study (DEPSY)’, in which a total of six child and adolescent psychiatric clinics in Germany joined forces [18]. However, for this analysis, only local data were used. Patients aged 0.31 to 5.95 years were included at the time of their initial presentation. The legal guardians completed the ACEs questionnaire (as well as other questionnaires; see Bödeker et al., 2023 [18]) and were supervised by the study physicians or study psychologists.

Only fully completed questionnaires were included in the evaluation. Of the original 127 legal guardians, 6 were excluded during the course of the study because they withdrew prematurely. Another 2 cases had to be excluded due to incomplete data, resulting in a sample of 116 participants (Table 1).

The data analysis included 116 legal guardians of patients. Between May 2022 and November 2024, 95 (81.9%) biological mothers, 17 (14.7%) biological fathers, 2 (1.7%) adoptive mothers, and 2 (1.7%) foster mothers completed the ACE questionnaire. In total, 83 (71.6%) boys and 33 (28.4%) girls were included in the study. The average age of the participating children was 3.99 years (SD = 1.35, range = 0.31–5.95 years) (Table 2).

The largest age group consisted of children in their fifth year (48–59 months) (*n* = 35, 30.2%), followed by those in their sixth year of life (60–71 months) (*n* = 29, 25%). The least represented age category was 0–11 months (*n* = 4, 3.4%).

Study procedure. All children aged 0.31 to 5.95 years who had an appointment at the special outpatient clinic for infants, toddlers, and preschool children in Homburg were eligible to participate in this study. Along with the invitation to the appointment, all caregivers received a clinical questionnaire that included questions about their family and developmental history, the adverse childhood experiences (ACEs) questionnaire, and either the Child Behavior Checklist (CBCL = 1.5–5) (for children aged 18 months and older) or the Questionnaire for Crying, Feeding, and Sleeping (QCFS) (for children under 18 months).

At the appointment, the parents were informed about the study and asked to give their consent. After signing the consent form, they received additional questionnaires that were developed specifically for the DePsy study.

Diagnoses were made using clinical criteria according to the International Classification of Diseases, 10th Revision (ICD-10) (published by the World Health Organization) and Diagnostic Classification of Mental Health and Developmental Disorders of Infancy and Early Childhood (DC:0–5) [19]. DC:0–5 is currently the most specific classification system for diagnosing mental disorders in young children aged 0 to 5 years, which is why we used these diagnoses here [20]. In addition, a standardized survey form was completed by the treating psychotherapist or study physician. In addition to the family and pregnancy history and existing support, this form also included the DC:0–5 and ICD-10 diagnoses. There was no time limit for completing the documents, and caregivers were free to ask questions at any time. They could complete the questionnaires immediately after their appointment at the clinic or at home, either on paper or online via the REDCap data system, which was accessible via an individual password.

Data collection for this study was approved by the Ethics Committee of the Saarland Medical Association (Saarland Medical Association Bu 15/22; date 31 January 2022).

Materials and methods. Sociodemographic data such as age and gender were collected and described. The Adverse Childhood Experiences Questionnaire (ACE-D) was used to record the parents’ stressful childhood experiences [1,21]. The questionnaire consists of 10 items that ask parents to retrospectively assess their experiences in the categories “abuse”, “family risk factors”, and “neglect”. It takes approximately 5 min to complete. A total of four or more ACEs is associated with increased behavioral problems in children [3]. In addition, higher exposure to adverse childhood experiences is associated with greater demand for coping mechanisms for mood regulation. Affected people are more likely to live unhealthily and develop a life with various risk factors such as smoking, alcoholism, drug abuse, depression, and suicide attempts, which, in turn, lead to related diseases such as diabetes, chronic bronchitis, and hepatitis. This behavior is unfavorable for the cardiovascular system. This vicious circle contributes to the development of multiple risk factors and increased health risks that should be recognized as the basic causes of morbidity and mortality in adulthood [1].

The children’s diagnoses were assigned using the DC:0–5, a revised classification of the DC0-3R, which covers early childhood mental disorders [22]. It was published in 2016, is largely based on the DSM-5, and is currently the most specific classification system for diagnosing mental disorders in young children [18,20].

Statistical analysis. The analysis was performed using the 29th version of IBM SPSS. First, descriptive statistics (means, standard deviations, frequencies, and percentages) were calculated to characterize the sample (Table 2). The prevalence of the individual ACEs, ACE categories, the number of ACEs and DC:0–5 diagnoses were summarized descriptively (Table 3, Table 4 and Table 5). To analyze the associations between parental ACE categories and children’s diagnoses (Table 6), odds ratios (ORs) with 95% confidence intervals (CIs) were calculated. For all analyses, ACEs were operationalized both as continuous sum score and as categorical variables.

## 3. Results

The average number of ACEs reported in the present study was 2.38 (SD = 2.45 [1,9]). The 17 biological fathers had an average value of 1.59, while the 95 participating mothers had an average value of 2.59. The two participating foster mothers had a value of 1, while the two adoptive mothers had the lowest average value of 0.5.

Table 3 shows the prevalence of individual ACEs in the study population as compared to the general population according to Witt et al., 2019 [2]. The most common reason reported was separation from a parent, cited by 48 participants (41.4%). By age 18, 29.4% (*n* = 5) of fathers and 43.2% (*n* = 41) of biological mothers had experienced parental absence due to divorce, abandonment, or other reasons. In the general population, this category was also described by Witt et al. as the most common, corresponding to a percentage of 19.4 [2]. The second most common factor, reported by 34.5% or 40 participants, was that a member of their household had a mental illness; this was followed by emotional abuse, which was experienced by 37 guardians (31.9%). Thirty-five (30.2%) participants classified themselves as having experienced emotional neglect, and the same number reported experiencing substance abuse by a household member.

**Table 3:** Overall prevalence of individual adverse childhood experiences (ACEs) among guardians in our study population, compared to the general population as described by Witt et al. [2].

ACE Category	Total (*n* Yes; % Yes)	Witt et al. (2019) (Table 2) [2]
ACE 1: Emotional abuse	37 (31.9%)	311 (12.5%)
ACE 2: Physical abuse	28 (24.1%)	230 (9.1%)
ACE 3: Sexual abuse	17 (14.7%)	109 (4.3%)
ACE 4: Emotional neglect	35 (30.2%)	338 (13.4%)
ACE 5: Physical neglect	10 (8.6%)	109 (4.3%)
ACE 6: Separation from a parent	48 (41.4%)	488 (19.4%)
ACE 7: Violence against mother	16 (13.8%)	248 (9.8%)
ACE 8: Substance abuse by a household member	35 (30.2%)	421 (16.7%)
ACE 9: Mental illness of a household member	40 (34.5%)	267 (10.6%)
ACE 10: Prison stay of a household member	10 (8.6%)	88 (3.5%)

In total, 68.1% of the respondents reported having experienced at least one adverse childhood experience by the age of 18, and the remaining 31.9% reported none. Among the guardians, 15.5% reported only one ACE, 12.1% reported two ACEs, and 10.3% reported three ACEs. The high-risk group with four or more ACEs comprised 30.2% of the participants (Table 4). In comparison, in the general population without traumatic childhood experiences, the largest category was those with one ACE at 20.7%.

**Table 4:** Prevalence of ACEs in categories of biological fathers and mothers, foster and adoptive mothers, and the total study population.

Number of ACEs	Total	Biological Father	Biological Mother	Foster Mother	Adoptive Mother
0	37 (31.9%)	6 (35.3%)	29 (30.5%)	1 (50%)	1 (50%)
1	18 (15.5%)	3 (17.6%)	14 (14.7%)	0	1 (50%)
2	14 (12.1%)	5 (29.4%)	8 (8.4%)	1 (50%)	0
3	12 (10.3%)	2 (11.8%)	10 (10.5%)	0	0
≧4	35 (30.2%)	1 (5.9%)	34 (35.8%)	0	0

The therapists and study doctors made confirmed diagnoses or expressed suspicion of a disorder in the infants. In all, 55 (47.4%) infants received one diagnosis, 22 (19%) received two diagnoses, 1 (0.9%) child received three diagnoses, and 2 (1.7%) received four diagnoses. A total of 18 different confirmed diagnoses were given.

Disorder of Dysregulated Anger and Aggression of Early Childhood formed the largest category. This diagnosis was made in 32 (27.6%) cases, and suspicion was expressed in another 19 (16.4%) patients. Twenty-one (18.1%) patients were diagnosed with global developmental delay, nineteen (16.4%) with an adjustment disorder, and eleven (9.5%) with attention deficit/hyperactivity disorder (Table 5). All other diagnoses occurred in less than 5% of cases.

**Table 5:** Diagnoses and suspected diagnoses of patients and average ACE scores of legal guardians for diagnoses.

	Diagnoses *	N (%) Confirmed	ACE Score	N (%) Suspected	ACE Score
4.2	Disorder of Dysregulated Anger and Aggression of Early Childhood	32 (27.6%)	2.47	19 (16.4%)	1.74
1.5	Global Developmental Delay	21 (18.1%)	2.24	-	-
9.2	Adjustment Disorder	19 (16.4%)	3.42	5 (4.3%)	1.2
1.3	Attention Deficit/Hyperactivity Disorder	11 (9.5%)	2.45	17 (14.7%)	0
6.2	Night-Waking Disorder	5 (4.3%)	2.8	5 (4.3%)	4.2
6.1	Sleep-Onset Disorder	4 (3.4%)	3.0	7 (6.0%)	3.71
3.1	Separation Anxiety Disorder	3 (2.6%)	2.67	2 (1.7%)	2.5
1.6	Developmental Language Disorder	3 (2.6%)	3.33	-	-
2.1	Sensory Over-Responsivity Disorder	2 (1.7%)	3.0	-	-
3.2	Social Anxiety Disorder (Social Phobia)	2 (1.7%)	5.5	4 (3.4%)	0.75
1.7	Developmental Coordination Disorder	1 (0.9%)	5.0	2 (1.7%)	3.5
3.3	Generalized Anxiety Disorder	1 (0.9%)	0	1 (0.9%)	1
7.2	Undereating Disorder	1 (0.9%)	1.0	-	-
8.1	Excessive Crying Disorder	1 (0.9%)	1.0	-	-
8.2	Other Sleep, Eating, and Excessive Crying Disorder of Infancy/Early Childhood	1 (0.9%)	4.0	1 (0.9%)	2
9.5	Disinhibited Social Engagement Disorder	1 (0.9%)	6.0	-	-
9.6	Other Trauma, Stress, and Deprivation Disorder of Infancy/Early Childhood	1 (0.9%)	0	-	-
10.1	Relationship-Specific Disorder of Infancy/Early Childhood	1 (0.9%)	2.0	-	-
3.7	Other Anxiety Disorder of Infancy/Early Childhood	-	-	3 (2.6%)	1.67
4.3	Other Mood Disorder of Early Childhood	-	-	3 (2.6%)	3.33
4.1	Depressive Disorder of Early Childhood	-	-	2 (1.7%)	1
9.1	Posttraumatic Stress Disorder	-	-	2 (1.7%)	1
1.2	Early Atypical Autism Spectrum Disorder	-	-	1 (0.9%)	5
1.4	Overreactive Disorder of Toddlerhood	-	-	1 (0.9%)	4.0
3.5	Selective Mutism	-	-	1 (0.9%)	3.0
9.4	Reactive Attachment Disorder	-	-	1 (0.9%)	0

* Five patients did not receive a diagnosis.

We analyzed these four most common diagnoses in relation to the parents’ average ACE scores. The legal guardians of children diagnosed with an adjustment disorder had the highest average ACE scores among the four most common diagnoses. Their score was 3.42, as compared to the average ACE score of 2.38 in our study population. This indicates that the caregivers of children with an adjustment disorder experienced more stressful childhood experiences than average. For the Disorder of Dysregulated Anger and Aggression of Early Childhood, the average ACE score was 2.47, slightly above the average of 2.38 in our study population; similarly, for attention deficit/hyperactivity disorder, the average score was 2.45. For global developmental delay, the score was 2.24, lower than our reference value. Among children with only suspected adjustment disorder (*n* = 5, 4.3%), the mean ACE score was 1.2. Among the 19 patients with suspected Disorder of Dysregulated Anger and Aggression of Early Childhood, the guardians had an average of 1.74 adverse childhood experiences. Seventeen (14.7%) of the children were diagnosed with attention deficit/hyperactivity disorder on suspicion, with their legal guardians scoring 2.53 on the ACE scale—similar to that observed with a confirmed diagnosis (2.45).

A statistical correlation with a *p*-value of 0.047 was found among children whose parents were affected by the ACE category of neglect (items 4 and 5) (Table 6). These children were 2.54 times more likely to have an adjustment disorder than children without parental neglect ACEs (OR = 2.54; 95% CI: 1.012–6.369; *p* = 0.047). For the ACE categories of maltreatment (OR = 1.49; *p* = 0.389) and family dysfunction (OR = 2.21; *p* = 0.125), there were no significant trends toward an increased risk. For children with other diagnoses, there was no significant association in any of the ACE categories. Here, the odds ratios were below one in some cases (ACE neglect: OR < 0.50).

**Table 6:** Odds ratios (ACE categories and adjustment disorder or other diagnoses).

ACE Category	Adjustment Disorder *n* (%) OR (95%-CI) *p*	Other Diagnoses * n (%) OR (95%-CI) *p*
ACE—Maltreatment (Items 1–3)	12 (24.5%) 1.49 (0.603–3.664) 0.389	36 (73.5%) 0.87 (0.372–2.027) 0.745
ACE—Neglect (Items 4 and 5)	12 (31.6%) 2.54 (1.012–6.369) 0.047	25 (65.8%) 0.50 (0.209–1.181) 0.113
ACE—Family Dysfunction (Item 6–10)	18 (25.4%) 2.21 (0.802–6.075) 0.125	50 (70.4%) 0.52 (0.205–1.290) 0.157
0–3 ACEs	15 (18.5%) 0.66 (0.256–1.686) 0.382	62 (76.5%) 1.31 (0.533–3.196) 0.560
≧ACEs	9 (25.7%) 1.52 (0.593–3.910) 0.382	25 (71.4%) 0.77 (0.313–1.876) 0.560

* In “other diagnoses”, we included all diagnoses that can be found specifically in Table 5, excluding adjustment disorder.

## 4. Discussion

To the best of our knowledge, this is one of the first studies to examine the transgenerational transmission of parental ACEs to preschool children in an early childhood mental health clinic.

The vast majority of parents (68.9%) of our early childhood patient population reported at least one ACE, which is substantially higher than the 43.7% reported in the general German population.

In the representative study by Witt et al., 43.7% of respondents reported having experienced at least one form of childhood trauma (≥1 ACE) [2]. While the proportion of individuals with four or more ACEs was 8.9%, the authors did not provide explicit information on the proportion with ≥2 ACEs. However, the latent class analysis reported in the same article suggests that approximately 22.2% of respondents were assigned to one of the three risk groups (“household dysfunction”, “child abuse and neglect”, or “polyadversity”), which are typically associated with multiple ACEs. It can therefore be assumed that about one-fifth of the general population reported ≥2 ACEs.

In our clinical sample of 116 parents of children attending a toddler clinic, the proportion of those with ≥2 ACEs was 52.6%. Broken down by gender, this was 53.5% (53 out of 99) of mothers and 47.05% (8 out of 17) of fathers. This shows an increased proportion with multiple adverse childhood experiences compared to the general population. In particular, the biological mothers of the patients reported, on average, one adverse childhood experience more than the biological fathers who participated in our study, although significantly fewer fathers participated.

These data and findings correspond to the prevalences reported by Altpeter et al. (2024) in a population of older child psychiatric patients [15]. While the age range of the children in the sample studied by Altpeter et al. (2024) [15] was between 6 and 18 years, the children in the sample studied here were between 0 and 5 years old.

For the most common diagnosis, Disorder of Dysregulated Anger and Aggression of Early Childhood, the mean ACE score of the parents was 2.47, clearly higher than that of the German general population (1.03). The ACE scores of parents of children with ADHD (2.45), general developmental delay (2.75), and adjustment disorders (3.42) were similarly high.

Intergenerational transmission could be mediated via parental psychopathology (depression, anxiety), an insecure attachment style, dysfunctional parenting, and stress reactivity—factors that can promote stress-related adjustment disorders in children [23]. Epigenetic mechanisms (e.g., altered DNA methylation, HPA axis dysregulation) might also contribute to vulnerability [10,24]. For instance, postmortem analyses of hippocampal tissue from adults with documented history of childhood abuse reveal decreased expression of the NR3C1 gene, which is thought to be involved in regulation of the HPA axis, along with increased DNA methylation in its regulatory region [25]. This suggests that childhood maltreatment may contribute to lasting epigenetic alterations in stress-regulatory genes, potentially affecting the function of the hypothalamic–pituitary–adrenal (HPA) axis across generations Lewis 2020.

Our findings are of considerable clinical relevance, as intergenerational transmission of trauma has been described and may be mediated both through interaction [26,27] and through neurobiological changes, endocrinological transmission, or even epigenetic characteristics.

Our data suggest that children of parents with ACEs should be considered for psychosocial or even clinical support as early as preschool age.

Increased stress in parents is associated with dysfunctional attachment behavior and inadequate emotion regulation towards their children [28,29]. In addition, lower parental sensitivity and a higher likelihood of inconsistent parenting are risk factors for childhood adjustment disorders [30].

It is not yet clear whether early parental care directly influences the brain structures that are important for emotional development, or whether it provides a social framework in which the child learns to deal with feelings and regulate their reactions. It is possible that the way parents interact with their child influences their stress system (e.g., hormonal responses to stress). This, in turn, could promote the development of brain regions, particularly in the frontal lobe, that are important for executive functions (such as attention, impulse control, and emotion regulation). This may create a positive cycle in which good parental care supports self-regulation in the child, which facilitates the relationship, which, in turn, further supports development [28].

At this age, the prognosis for interaction therapy is therefore particularly favorable, as dysfunctional relationship patterns have not yet become ingrained and chronic. This underscores the importance of offering comprehensive support services for parents of young children, with the option of interaction therapy. In addition, it is important for early childhood services to be aware that parents represent a more vulnerable risk group than the general population and that services for young children should therefore address not only the children but also the therapeutic needs of their parents.

New structures must be created for this purpose, as our data underscore.

We have refrained from describing the average ACE scores for all individual diagnoses of the children. For groups with fewer than 10 cases, the mean value can only be interpreted to a limited extent due to the small number of cases. For a better interpretation of this correlation, we would recommend a further study with a higher number of cases.

Limitations: The interpretation of our findings is constrained by the cross-sectional design, which does not allow causal inferences. While the observed associations between parental ACEs and children’s psychosocial outcomes are noteworthy, directionality cannot be established. As Parental ACEs date back to the caregiver’s childhood, it seems unlikely that diagnosis of the offspring may have influenced this variable. However, it is also possible that unmeasured factors, such as parental mental health, socioeconomic circumstances, or current family stressors, may have contributed to the associations we observed. Although information on socioeconomic status, parental education, and other contextual variables was available, the limited sample size did not allow for meaningful stratified or sensitivity analyses without producing unstable estimates and distorted results. Consequently, the potential influence of these contextual factors in shaping the findings cannot be ruled out. Future research with larger samples should therefore include socioeconomic and educational background more systematically, both descriptively and analytically, to enhance the interpretability and generalizability. In addition, longitudinal studies are needed to clarify temporal ordering, disentangle potential confounders, and better understand the mechanisms underlying intergenerational transmission of ACEs.

No trauma interview was conducted with the parents, and since the special consultation was offered for the children, no general psychodiagnostics interview of the parents took place. The diagnoses of the children according to DC:0–5 were assigned according to the clinical criteria in the German-language manual [19]. The sample size of 116 is not large, but it is sufficient to demonstrate moderate effects. However, the relatively small sample size could restrict the statistical power and limit the generalizability of the findings. As the sample was recruited from a clinic-referred population, selection bias cannot be ruled out. The cross-sectional design precludes conclusions about causal relationships; prospective or longitudinal data would be necessary to clarify the temporal dynamics between parental ACE and child psychopathology. Furthermore, given the lack of prior evidence on the differential transmission of parental ACEs to specific forms of preschool psychopathology, this study should be regarded as exploratory and hypothesis-generating rather than confirmatory. Moreover, there are several other possible moderators and mediators (e.g., sociodemographic: gender, age; psychological: attachment, temperament, impulsivity, resilience; other stressful illnesses) in the association between ACEs and childhood diagnoses, which should be taken into consideration by future work.

Clinical relevance: Our study advocates for an early and comprehensive psychological and medical history assessment, i.e., recording not only the child’s current environment and behavioral problems but also the childhood experiences of the parents and, if applicable, the grandparents. Risk constellations can thus be identified earlier in the anamnesis and through parent–child interaction diagnostics.

Furthermore, unresolved traumas experienced by parents can be transferred to their children. Parents with untreated ACEs are more likely to display disorganized attachment styles, which influences the quality of their interaction with their children and can contribute to mental health problems in the children. Clinical approaches should then focus on reflecting on the parents’ relationship experiences and promoting their sensitivity and responsiveness in parent–child interaction. Supplementary work with the parental subsystem is not an accessory but central to children with mental health issues and parental ACE stress. Our findings underscore the potential relevance of parental ACE for early childhood psychopathology. Future research should clarify underlying mechanisms and examine mediating or moderating factors, ideally using longitudinal and multilevel approaches. It should further investigate potential moderating variables that may shape the association between parental ACEs and child psychopathology, such as availability of social support networks or access to treatment services. Moreover, it will be important to disentangle specific patterns of ACE transmission—for instance, examining whether neglect and abuse exert differential effects on early childhood outcomes. Such investigations could provide more fine-grained insights into the mechanisms underlying intergenerational risk and help inform targeted prevention and intervention strategies.

## Figures and Tables

**Table 1:** Participant flow.

Phase	Number of Participants	Comment
Included	127	Parents who wanted to participate in the study
Consented	124	Consent form available
Excluded	8	Missing values
Included in the analysis	116	Complete data

**Table 2:** Description of the sample.

Variable	*n* (%)	Age M (SD) [Range]
Participant’s legal guardian		
Biological father	17 (14.7%)	39.47 (3.78) [32; 47]
Biological mother	95 (81.9%)	33.54 (5.01) [22; 43]
Foster mother	2 (1.7%)	*
Adoptive mother	2 (1.7%)	*
Participating patients		3.99 (1.35) [0.31; 5.95]
Boys	83 (71.6%)	4.11 (1.27) [0.60; 5.95]
Girls	33 (28.4%)	3.71 (1.53) [0.31; 5.92]
1st year of life, 0–11 months	4 (3.4%)	
2nd year of life, 12–23 months	7 (6.0%)	
3rd year of life, 24–35 months	14 (12.1%)	
4th year of life, 36–47 months	27 (23.3%)	
5th year of life, 48–59 months	35 (30.2%)	
6th year of life, 60–71 months	29 (25.0%)	

(M = mean; *n* = number of persons; SD = standard deviation; * age at birth was asked only of biological parents, not of foster and adoptive mothers).

## Data Availability

The original contributions presented in this study are included in the article; further inquiries can be directed to the corresponding author.
